# Characterization of Super-Responder Profile in Chronic Plaque Psoriatic Patients under Guselkumab Treatment: A Long-Term Real-Life Experience

**DOI:** 10.3390/jcm13175175

**Published:** 2024-08-31

**Authors:** Lorenzo Marcelli, Alfredo Belcastro, Marina Talamonti, Claudia Paganini, Angela Fico, Lorenzo Savastano, Cosimo Di Raimondo, Laura Vellucci, Luca Bianchi, Marco Galluzzo

**Affiliations:** 1 Department of Systems Medicine, University of Rome “Tor Vergata”, Via Montpellier 1, 00133 Rome, Italy; lmarcelli96@gmail.com (L.M.); belcastro.alfredo98@gmail.com (A.B.); cld.paganini@gmail.com (C.P.); angefico@live.it (A.F.); lolonzo@hotmail.it (L.S.); vellucci.laura@gmail.com (L.V.); luca.bianchi@uniroma2.it (L.B.); 2 Dermatology Unit, Fondazione Policlinico “Tor Vergata”, 00133 Rome, Italy; talamonti.marina@gmail.com (M.T.); cosimodiraimondo@gmail.com (C.D.R.)

**Keywords:** psoriasis, super responder, guselkumab, bio-naïve

## Abstract

**Background:** The term “super responder” identifies a group of patients who exhibit a rapid and optimal response to biological treatment compared to the overall treated population. The primary objective of our study is to characterize this subgroup of patients to enable the early identification of those who will respond most effectively to the proposed treatment while also evaluating clinical efficacy. **Methods:** This retrospective study evaluated 232 patients treated with guselkumab in monotherapy for at least 20 weeks between November 2018 and November 2023. Patients were divided into two groups: those who achieved complete clear skin (PASI = 0) during the first 20 weeks of treatment were defined as super responders (SRe) and non-super responders (nSRe) were the remaining patients. PASI was assessed at weeks 0, 4, and subsequently every eight weeks. Predictors of the SRe status were evaluated by univariate and multivariate logistic regression analyses. **Results:** The univariate analyses showed that patients with psoriatic arthritis at the baseline, bio-naïve patients, or those who had not received an interleukin (IL) 17 inhibitor as their last therapy before guselkumab administration were more likely to be super responders to the proposed treatment. Multivariate logistic analysis models suggested that the combination of psoriatic arthritis at the baseline and the bio-naïve condition was the strongest predictive model for the SRe status. At week 204, the main difference between the two groups concerned the achievement of PASI100, maintained by 86.8 of SRe compared to 62.8% of nSRe. **Conclusions:** The efficacy and safety of guselkumab are confirmed in our real-life experience. Identifying the SRe status will undoubtedly play a role in clinical practice and the therapeutic decision-making algorithm.

## 1. Introduction

Psoriasis is a chronic inflammatory skin disease characterized by hyperproliferative keratinocytes and immune cell infiltration. Affecting approximately 2–3% of the global population worldwide, psoriasis significantly impairs quality of life due to its physical and psychological burden [1,2]. The pathogenesis of psoriasis involves complex interactions between genetic predispositions, environmental factors, and immune dysregulation [3]. 

Recent advancements in understanding the immunological pathways of psoriasis have highlighted the critical role of the IL-23/Th17 axis [1]. IL-23 was identified in 2000 as a heterodimer composed of the IL-12/23p40 subunit and a newly discovered p19 subunit that is exclusive to IL-23 [4]. IL-23 is a ‘master regulator’ cytokine in many chronic inflammatory diseases, particularly in psoriasis. IL-23 bridges the innate and adaptive immune systems: it acts on T cells as well as innate immune cells (e.g., natural killer cells, macrophages, dendritic cells, and innate lymphoid cells), normally confers immunity against bacterial and fungal infections, and it is pivotal in maintaining and expanding Th17 cells, which produce pro-inflammatory cytokines such as IL-17A, IL-17F, and tumor necrosis factor (TNF) [5,6]. These cytokines are essential for the psoriatic inflammatory cascade, promoting keratinocyte proliferation and inflammation [3]. Targeting this axis has led to the development of highly effective biologics, including IL-17 and IL-23 inhibitors, revolutionizing psoriasis treatment [1].

Guselkumab, a fully human monoclonal antibody targeting the p19 subunit of IL-23, has shown remarkable efficacy in clinical trials. It significantly reduces Psoriasis Area and Severity Index (PASI) scores and maintains long-term skin clearance with a favorable safety profile [7]. It was approved in 2017 by the European Medicines Agency (EMA) for the treatment of moderate-to-severe chronic plaque psoriasis and in 2020 for the treatment of psoriatic arthritis. It has been available since October 2018 in Italy. From the phase III development program of guselkumab in plaque psoriasis, the VOYAGE-1 trial, in particular, demonstrated that guselkumab achieved a PASI75 in 91%, a PASI90 in 76.3%, and a PASI100 response in 47.4% of patients at week 48, outperforming other treatments in maintaining long-term skin clearance [1,7,8].

The efficacy of guselkumab was maintained in the open-label extension periods through week 252 in VOYAGE 1 and VOYAGE 2 trials: 84.1% of patients in VOYAGE 1 and 82% of those in VOYAGE 2 achieved PASI 90 at week 252 in the guselkumab group and, furthermore, 52.7% of patients in VOYAGE 1 and 53% of those in VOYAGE 2 achieved PASI 100 [9,10].

Previous studies have demonstrated that a rapid achievement of PASI90 during treatment with biological drugs is a predictive factor for increased drug survival [11,12]. In particular, GUIDE (NCT03818035) is a phase 3b, randomized, double-blind, multi-center study (80 centers in Germany and France) that has included more than 850 adult patients affected by moderate-to-severe plaque-type psoriasis and explored if early intervention in psoriasis with the IL-23 inhibitor guselkumab may lead to long-lasting and disease-modifying effects [13]. In GUIDE part 1 (week [W0–W28]), patients received guselkumab, 100 mg, at W0, W4, W12, and W20 and were qualified as “super responders (SRes)” if they achieved a Psoriasis Area and Severity Index (PASI) score of 0 at both W20 and W28. In part 2 (W28–W68) of the study, the primary objective was to demonstrate the noninferiority (with a 10% margin) of guselkumab dosing every 16 weeks vs. every 8 weeks among SRes for the maintenance of disease control. The preliminary results of this study at week 28 characterized SRe patients as younger, with a shorter disease duration, who more often had a BMI < 25 kg/m^2^, less frequently had nail involvement, and were more likely to be bio-naïve compared to non-SRe patients [14].

In part 2, the primary endpoint of the noninferiority of guselkumab dosing every 16 weeks vs. every 8 weeks for the maintenance of disease control in SRes was met, with a PASI score of less than 3 at 68 weeks achieved by 137 of 149 patients treated with guselkumab every 16 weeks (91.9%; 90% CI, 87.3–95.3%) and 137 of 148 patients treated with guselkumab every 8 weeks (92.6%; 90% CI, 88.0–95.8%; odds ratio [OR] 0.92; 90% CI, 0.45–1.87; *p* = 0.001 for noninferiority). SRes maintained high rates of a PASI of 1 or lower and a PASI of 0 response over time and at W68, with higher response rates with dosing every 8 weeks vs. every 16 weeks (PASI ≤ 1 at W68: 133/148 every 8 weeks [89.9%], 118/149 patients [79.2%] dosed every 16 weeks; *p* = 0.01; PASI = 0 at W68: 120/148 every 8 weeks [81.1%], and 103/149 patients [69.1%] dosed every 16 weeks; *p* = 0.02) [15].

Building on the GUIDE study results, we aimed to characterize the profile of SRe patients in an Italian real-life setting. Identifying the SRe status will undoubtedly play a role in clinical practice and the therapeutic decision-making algorithm, ensuring rapid and lasting therapeutic responses from the beginning, with high drug survival rates and the possibility of extending the dosing interval.

## 2. Materials and Methods

### 2.1. Study Design

This is a retrospective observational, cross-sectional, “snapshot”, single-center study conducted at the Dermatology Unit of the Policlinico Tor Vergata. We reviewed data of adult patients (≥18 years old) who were receiving monotherapy treatment with guselkumab, affected by moderate–severe chronic plaque psoriasis who were treated for at least 20 weeks (three doses) and who had started treatment between 1 November 2018 and 31 November 2023. 

Administration of guselkumab followed the Summary of Product Characteristics (briefly, an induction with a subcutaneous administration of 100 mg at weeks 0 and 4 and a maintenance administration every 8 weeks) including patients who did not respond to or showed contraindications or side-effects to at least one conventional systemic therapy and with a: (i) PASI ≥ 10 and Body Surface Area (BSA) ≥10 at baseline; or (ii) PASI < 10 and BSA < 10 at baseline, as well as involvement of specific sensitive areas (i.e., face, scalp, hands, nails, soles of the feet, or genital areas), according to Italian law.

Patients were excluded from the study if they were receiving any co-medication with systemic or topical therapies (topical corticosteroids, vitamin D derivatives).

Exclusion criteria included patients with other autoimmune/inflammatory diseases such as Crohn’s disease, ulcerative colitis, rheumatoid arthritis, and ankylosing spondylitis, patients with active infections, pregnant or breastfeeding patients, patients treated with a biologic < 4 weeks or patients that had received systemic treatment or phototherapy in combination with biologic agent within 4 weeks of the first visit, and patients with guttate, erythrodermic, or pustular psoriasis. 

At baseline (T0), all patients underwent thorough screening investigations to assess their eligibility for guselkumab treatment. In particular, Quantiferon-TB gold tests and serology for hepatitis B virus (HBV), hepatitis C virus (HCV), and human immunodeficiency virus (HIV), hematology panel, serum chemistry panel (glucose, transaminases, creatinine, blood urea nitrogen, total protein, total and fractionated bilirubin, lactate dehydrogenase, creatine phosphokinase, gamma-glutamyl transferase, and alkaline phosphatase), anti-nuclear antibodies, extractable nuclear antigens, and urinalysis panel. 

Patients started treatment at different times during the study, so these data represent only a cross-sectional ‘snapshot’ of our experience taken up to the end of April 2023.

PASI evaluation was conducted at baseline, at week 4 of treatment, and subsequently every eight weeks. Clinical efficacy was assessed using PASI90 and PASI100. 

The primary endpoint of the study was to characterize SRe patients in a real-life context to identify early on the profile of patients who will respond most effectively to the proposed treatment. Patients who achieved a PASI = 0 by week 20 were considered SRe. The secondary endpoint was the evaluation of long-term clinical efficacy. 

Ethical approval for this study was obtained from “COMITATO ETICO INDIPENDENTE FONDAZIONE POLICLINICO TOR VERGATA” (Registro Sperimentazioni 46.23—Parere 31 March 2023).

### 2.2. Statistical Analysis

Data are presented as mean ± standard deviation for continuous variables and as numbers and percentages for categorical variables. Univariate logistic regression analysis was performed considering sex, age, age of disease onset, disease duration, weight, body mass index (BMI), baseline PASI score, presence of psoriatic arthritis (PsA) at baseline, number of comorbidities, number of previous biologics, last class of biologics used, and presence of disease in difficult-to-treat sites to evaluate a possible association with the SRe status. Stepwise multivariate logistic regression models were performed to determine the best predictive models that can define a patient as SRe to guselkumab using the same variables analyzed in the univariate analysis.

If any study values were not recorded for an intermediate patient visit, the value was assigned using the last observation carried forward (LOCF) method. Statistical significance was set at *p*-value < 0.05. All analyses were performed using STATA 11.2 software (StataCorp LP Inc., College Station, TX, USA).

## 3. Results

### 3.1. Patients

The data of 232 patients treated with guselkumab monotherapy for at least 28 weeks between November 2018 and November 2023 were collected. At the time of the data analysis, 181 out of 232 patients had reached week 32 of treatment; 161 had reached week 66; 144 had reached week 100; 114 had reached week 124; 90 had reached week 156; and 66 had reached week 204. 

The mean age of the study population was 54.3 years (SD 14.7) and 58.2% were males. The mean disease duration was 25.4 years (SD 15.3) with a mean disease onset of 28.9 years (SD 16.6) and the mean body mass index (BMI) was 28.1 kg/m^2^ (SD 5.7). In addition, 68% of patients had received prior biological therapy (94 patients had received one previous biologic, 37 had received two, 17 patients had received three, 5 patients had received four, 3 patients had received five, and 1 patient had received six). Obesity and hypertension were the most frequent comorbidities observed (31.9% and 29.8%, respectively). In total, 26 patients had a concomitant diagnosis of psoriatic arthritis at the baseline. The demographic and clinical characteristics of the study population are reported in Table 1.

Additionally, 144/232 (62%) patients who achieved complete clear skin (PASI = 0) by week 20 of treatment were defined as super responders (SRe). The remaining patients were classified as nSRe (88/232 (38%)). The clinical and demographic characteristics were found to be similar between the two groups (SRe vs. nSRe) in terms of the mean age (53.9 vs. 54.9 years), age of psoriasis onset (29.6 vs. 27.8), disease duration (24.1 vs. 27 years), BMI (27.8 vs. 28.7 kg/m^2^), and baseline PASI score (14.1 vs. 13.5).

SRe patients were more often bio-naïve compared to nSRe patients (36.8% vs. 25%).

### 3.2. Univariate and Multivariate Logistic Regression Analysis 

A univariate logistic regression analysis was carried out considering sex, age, age of disease onset, disease duration, weight, BMI, PASI score at the baseline, number of comorbidities, number of previous biologics, last class of biologics drug used, and presence of disease in a difficult-to-treat area (Table 2).

Univariate logistic regression showed that psoriatic arthritis at the baseline was a positive predictive factor for super-responder status at week 20 (OR 2.83, *p*-value 0.044). Also, being bio-naïve was significantly associated with a better probability of achieving SRe status (OR 1.85, *p*-value 0.040). Specifically, the IL-17 inhibitor as the last therapy before guselkumab administration negatively affected the status of super responders (OR 0.53, *p*-value 0.043). Regarding previous therapy before guselkumab, switching from other biologic drugs did not show statistically significant data. On the other side, the mean age (OR 0.99, *p*-value 0.544), age of psoriasis onset (OR 1.01, *p*-value 0.516), disease duration (OR 0.99, *p*-value 0.199), BMI (OR 0.97, *p*-value 0.270), baseline PASI (OR 1.01, *p*-value 0.603), and involvement of special sites, such as scalp (OR 1.45, *p*-value 0.185), genitals (OR 1.13, *p*-value 0.0692), nails (OR 0.77, *p*-value 0.0582), and palmoplantar (OR 0.66, *p*-value 0.324) did not influence the chance to reach SRe status.

A multivariate analysis showed that the models most frequently associated with the SRe status were patients with concomitant PsA at the baseline (OR 2.93, *p*-value 0.039) and a bio-naïve status (OR 1.9, *p*-value 0.035) (Table 3), patients with concomitant PsA at the baseline (OR 3.64, *p*-value 0.017) and a lower number of previous biological therapies (OR 0.75, *p*-value 0.023) (Table 4), and patients with concomitant PsA at the baseline (OR 3.07, *p*-value 0.034) who did not have the IL-17 inhibitor as their last therapy before guselkumab (OR 0.46. *p*-value 0.019) and did not have anti-TNFα as their last therapy before guselkumab (OR 0.42, *p*-value 0.042) (Table 5).

### 3.3. Long-Term Clinical Response

**Figure 1:**
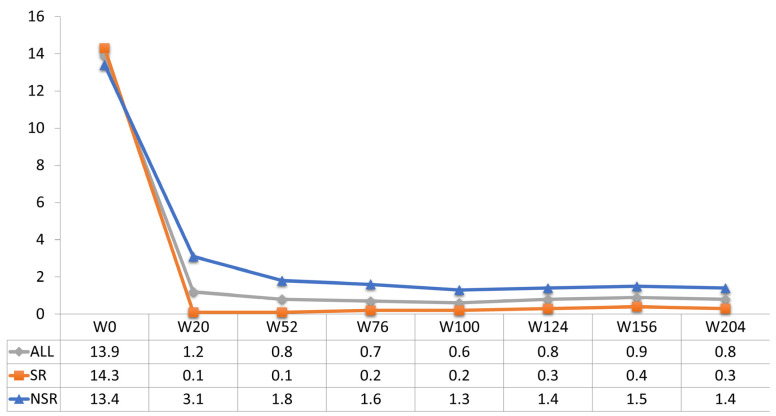
Mean PASI score up to week 204 ITT-LOCF (intention to treat—last observation carried forward).

**Figure 2:**
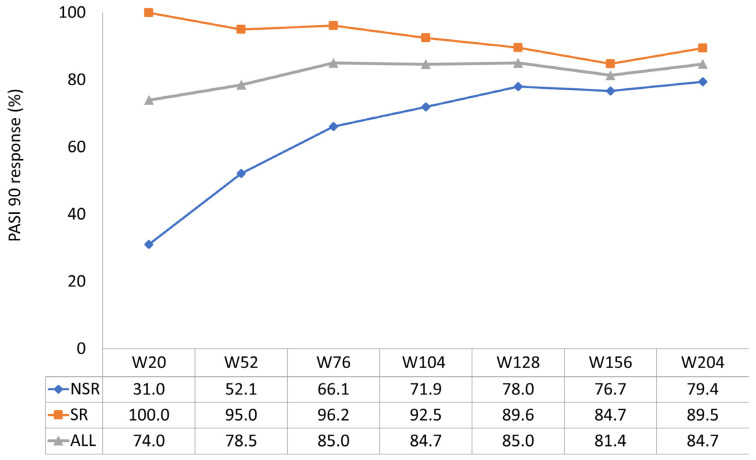
PASI90 achievement up to week 204 (ITT-LOCF).

**Figure 3:**
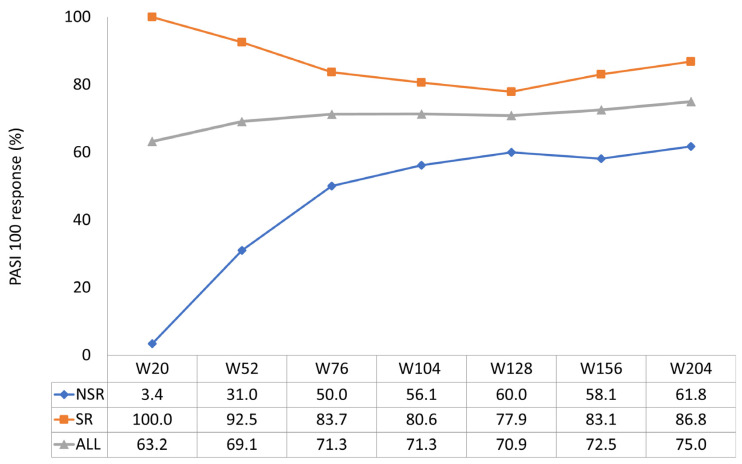
PASI100 achievement up to week 204 (ITT-LOCF).

The effectiveness of guselkumab was evident in the short term, as shown by the average PASI reduction graph. Guselkumab therapy demonstrated significant efficacy in reducing the mean PASI in the entire study population, from 13.9 at the baseline to 1.2 at week 20, 0.8 at week 52, and 0.8 at week 124, a value maintained up to week 204 (Figure 1). The speed of action of guselkumab was higher in SRes (reduction of a mean PASI from 14.3 at the baseline to 0.1 at week 20, maintained to week 52) than in nSRe. However, differences in the percentage rates of achieving PASI 90 and PASI 100 between the SRe and nSRe groups were noted in the long term. At week 52, 95% of SRe patients achieved PASI90 compared to 52.1% of nSRe patients. By week 204, the difference in achieving PASI90 between the two groups was reduced (89.5% SRe vs. 79.4% nSRe) (Figure 2). The most significant difference was observed in achieving PASI100 both in the short and long term. At week 52, 92.5% of SRe patients maintained PASI100, while only 31% of nSRe patients achieved complete skin response in the same week. After four years of treatment, 86.8% of SRe patients maintained a condition of completely clear skin compared to 61.8% of nSRe patients (Figure 3).

### 3.4. Safety and Drop-Out

No adverse events of interest related to the drug were observed during the observation period. In particular, none of the 21 patients with latent tuberculosis at the baseline had developed active tuberculosis. No cutaneous or mucosal fungal infections were recorded. Fourteen patients (6.03%) discontinued guselkumab therapy. Of these, only 4 were from the SRe group. In 3 patients, discontinuation was due to adverse events, and in 1 patient, it was due to the desire for pregnancy. In all four patients, the PASI at the time of discontinuation was 0. In 8 out of 10 nSRe patients who discontinued therapy, the reason for discontinuation was a secondary loss of efficacy.

## 4. Clinical Case of a Super-Responder Patient

**Figure 4:**
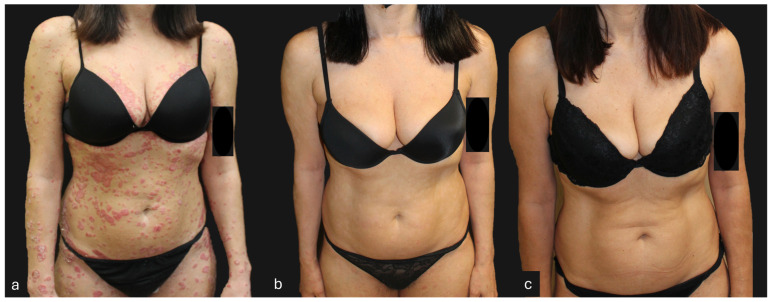
Patient with a super-responder profile treated with guselkumab (**a**) at baseline (PASI score = 50), (**b**) after 12 weeks of treatment (PASI score = 0), and (**c**) after 156 weeks of treatment (PASI score = 0). The patient, after 104 weeks of treatment, received guselkumab every 12 weeks instead of the standard guselkumab treatment regimen (every 8 weeks).

**Figure 5:**
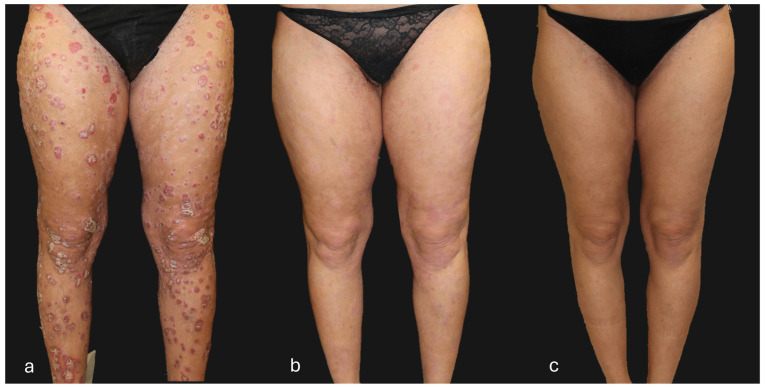
Patient with a super-responder profile treated with guselkumab (**a**) at baseline (PASI score = 50), (**b**) after 12 weeks of treatment (PASI score = 0), and (**c**) after 156 weeks of treatment (PASI score = 0). The patient, after 104 weeks of treatment, received guselkumab every 12 weeks instead of the standard guselkumab treatment regimen (every 8 weeks).

**Figure 6:**
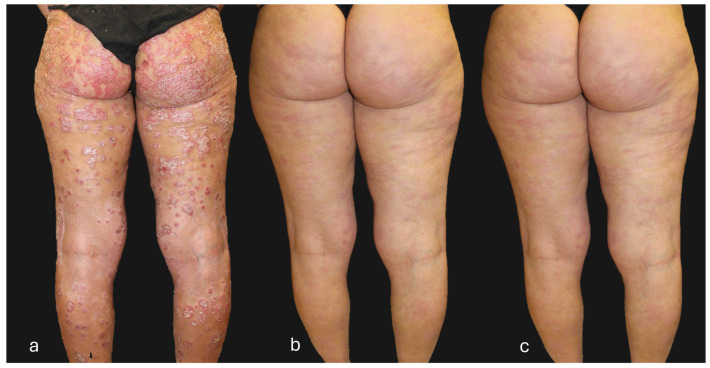
Patient with a super-responder profile treated with guselkumab (**a**) at baseline (PASI score = 50), (**b**) after 12 weeks of treatment (PASI score = 0), and (**c**) after 156 weeks of treatment (PASI score = 0). The patient, after 104 weeks of treatment, received guselkumab every 12 weeks instead of the standard guselkumab treatment regimen (every 8 weeks).

**Figure 7:**
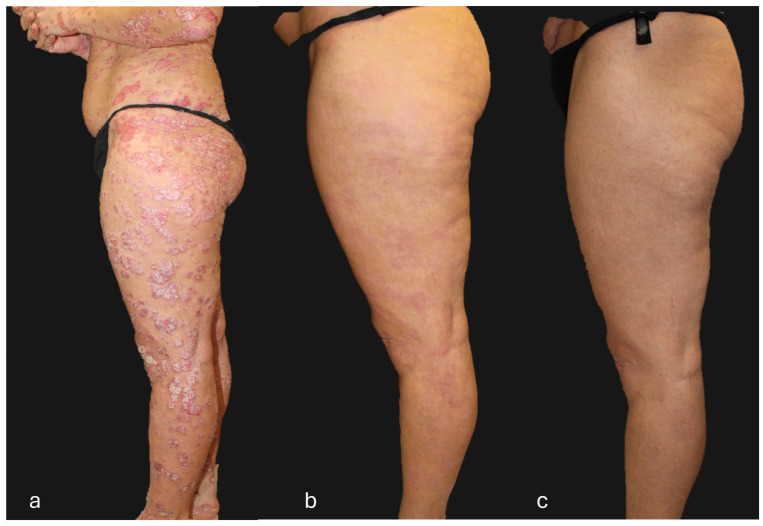
Patient with a super-responder profile treated with guselkumab (**a**) at baseline (PASI score = 50), (**b**) after 12 weeks of treatment (PASI score = 0), and (**c**) after 156 weeks of treatment (PASI score = 0). The patient, after 104 weeks of treatment, received guselkumab every 12 weeks instead of the standard guselkumab treatment regimen (every 8 weeks).

**Figure 8:**
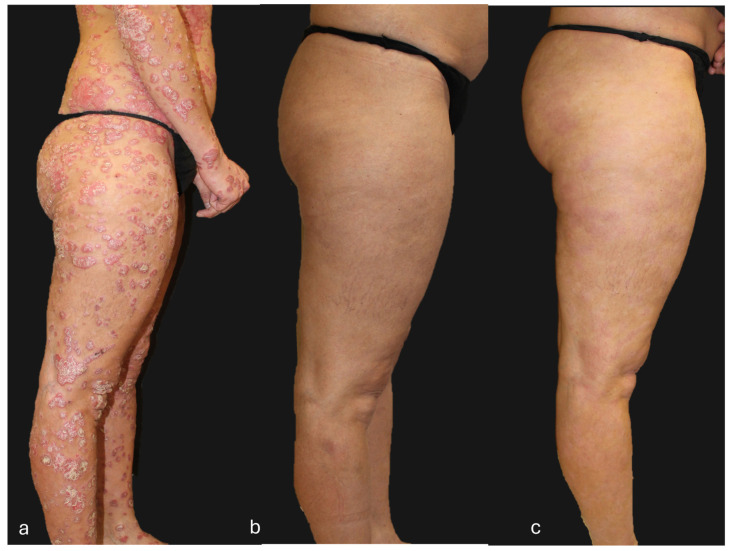
Patient with a super-responder profile treated with guselkumab (**a**) at baseline (PASI score = 50), (**b**) after 12 weeks of treatment (PASI score = 0), and (**c**) after 156 weeks of treatment (PASI score = 0). The patient, after 104 weeks of treatment, received guselkumab every 12 weeks instead of the standard guselkumab treatment regimen (every 8 weeks).

A female patient, 47 years old, with chronic plaque psoriasis since the age of 14. Non-smoker, occasional alcohol consumption, with an anxiety disorder under pharmacological treatment. Previously treated with cyclosporine in 2013, this was discontinued due to poor compliance, biologic-naïve. In October 2020, she presented to our clinic with severe psoriasis, exhibiting a PASI score of 50 and involvement of difficult-to-treat areas such as the scalp, face, and palmoplantar regions. In December 2020, she started treatment with Guselkumab 100 mg. An excellent response was achieved within the first 12 weeks of treatment, reaching a PASI score of 0. Currently, the patient is at week 180 of treatment. Moreover, since week 104, the patient has received guselkumab every 12 weeks instead of every eight weeks, as indicated by the technical data sheet, maintaining complete clinical remission of the disease (Figure 4, Figure 5, Figure 6, Figure 7 and Figure 8).

## 5. Discussion

The efficacy and safety of guselkumab have already been confirmed by registered clinical studies and real-world evidence [16]. Our data on long-term effectiveness and safety are comparable, if not superior, to those obtained in guselkumab phase 3 clinical studies in chronic psoriasis VOYAGE1, VOYAGE2, and NAVIGATE, and in other real-life contexts [8,9,10,17,18]: at week 20 of our real-life experience, the rate of PASI90 response was 74%, while 63.2% of patients achieved completely clear skin (PASI100) in the same week. These percentages are higher than those observed in both VOYAGE1 and VOYAGE2 studies at week 24 and in the GUIDE study at week 28, where PASI100 was achieved in 44.4% of patients [8,9,10,13,14,15].

From our experience regarding the concept of super-responders to guselkumab therapy, which has been addressed exclusively in the GUIDE study so far, it emerges that this status is positively influenced by being bio-naïve and by the presence of PsA at the baseline and negatively by previous therapy with IL-17 inhibitors. The GUIDE study reports that super-responder patients tend to be younger, with a shorter disease duration, a BMI < 25 kg/m^2^, no nail involvement, and a bio-naïve status compared to the nSRe group [14]. Our real-life experience showed that the disease duration, patient age, BMI, and involvement of special sites did not impact the clinical response in the first 20 weeks. The impact of BMI on the clinical response had already been evaluated by our group in real-life, demonstrating the excellent efficacy of the drug in patients with severe obesity [19]. Our data confirm that being bio-naïve is a positive predictive factor for SRe status. This finding is consistent with analyses from other studies showing that being bio-naïve is a positive predictive factor for achieving PASI100 at all time-points [20,21]. 

The aim of our work was to identify patients who were super responders to guselkumab (achieving completely clear skin in the first 20 weeks of treatment). This does not mean that patients with some clinical characteristics (such as the involvement of difficult-to-treat areas) will not respond to the drug. Maybe this will happen after week 20, as we can see in the long-term data response.

The differences observed are likely due to the different baseline characteristics of the study populations considered, as the GUIDE phase IIIB study involved subjects selected with more stringent criteria. Regarding the long-term efficacy of guselkumab treatment, the entire study population observed an excellent response in terms of reducing the mean PASI and achieving PASI90 and PASI100. In addition to the GUIDE study findings, we highlight differences in the long-term response and drug survival between the two analyzed groups. As previously reported, the main differences between the two populations were observed in the rates of achieving and maintaining PASI100 (PASI score = 0 at week 204), which were substantially higher in the SRe group. Drug survival was also better in the SRe population, with four dropouts (2.8%) compared to 10 nSRe dropouts (11.4%). Being bio-naïve was one of the main predictive factors for super-responder status. This, combined with the increased drug survival observed in the SRe group, would justify the choice of guselkumab as a first-line systemic therapeutic option in moderate-to-severe psoriatic patients. Additionally, once a complete and prolonged response was achieved, some patients began extending the drug administration to every 12 weeks without experiencing disease recurrence. This category included 22% of SRe and 9% of nSRe. Although this observation does not reflect the EMA-recommended posology, it offers a significant advantage in pharmacoeconomics, warranting further investigation. In fact, the only available data come from Herranz-Pinto et al. and show that extending the interval between doses during the maintenance period does not compromise the efficacy of guselkumab therapy, particularly in bio-naïve and SRe patients [22].

The limitations of this study are mainly due to its retrospective nature and the fact that the patient population comes from a single study center. A multicenter study to expand the sample size is necessary to strengthen the obtained data. Currently, there are no data in the literature related to the potential correlation of the speed of response in terms of complete skin clearance and concomitant psoriatic arthritis in moderate-to-severe psoriatic patients. The only data available on this item come from Pirro et al., who considered patients treated with etanercept and adalimumab [23]. This finding also warrants further investigation and potential confirmation by analyzing a larger number of patients. 

## 6. Conclusions

Our real-life experience confirms the efficacy and safety profile of guselkumab shown in phase 3 clinical trials in chronic psoriasis, but it has the potential advantage of identifying a subgroup of patients with psoriasis treated with the IL-23 inhibitor guselkumab for whom it is possible to extend the dose administration without compromising clinical efficacy. While on-label dosing regimens involve injections every eight weeks, in fact, a considerable number of patients may successfully extend their dose administration intervals to 12 weeks for guselkumab. In particular, the most suitable candidates for extended dosing are those achieving complete skin clearance (Psoriasis Area and Severity Index = 0, body surface area = 0%) at week 20 of treatment who exhibit concomitant PsA at the baseline, are bio-naïve, or have not received an IL-17 inhibitor as previous biological therapy. Identifying the SRe status will undoubtedly play a role in clinical practice and the therapeutic decision-making algorithm. The better delineation of the SRe patient profile would ensure rapid and lasting therapeutic responses from the beginning, with high drug survival rates. The rationale behind extending the dosing schedule lies in early intervention with a selective and direct IL-23 inhibitor such as guselkumab, which specifically interferes with the major disease-driving pathway in psoriasis and could have the potential of restoring a ‘healthy’ Th17/Treg balance and controlling TRM levels, thus promoting a long-lasting and possibly disease-modifying therapeutic effect. Incorporating extended dosing intervals for IL-23 inhibitors in psoriasis management is in line with the growing emphasis on personalized medicine and patient-centered care and not only alleviates the treatment burden on patients, but also holds the potential to reduce healthcare costs and resource utilization.

At the current state, this represents the first evidence that in patients with early complete skin clearance at week 20, extending the guselkumab dosing interval may control thee disease activity.

## Figures and Tables

**Table 1:** Baseline clinical characteristics of psoriasis patients.

Clinical Characteristic	N = 232
General	
Male gender *n* (%)	135 (58.2)
Age (years)	54.3 (±14.7)
BMI (kg/m^2^)	28.1 (±5.7)
Current smoker, *n* (%)	115 (49.6)
Disease characteristics	
Age at disease onset (years)	28.9 (±16.6)
Disease duration (years)	25.4 (±15.3)
PASI at baseline	13.9 (±11.3)
Biologic therapy, *n* (%)	
Bio-naïve, *n* (%)	75 (32.3)
1 biologic	94 (40.5)
2 biologics	37 (15.9)
≥3 biologics	26 (11.2)
Comorbidities, *n* (%)	
Hypertension	69 (29.7)
Psoriatic arthritis	26 (11.2)
Obesity	74 (31.9)
Psychiatric illness	16 (6.9)
Thyroid dysfunction	17 (7.3)
Diabetes mellitus	28 (12.1)
TB GOLD positivity	21 (9.1)

BMI, body mass index; PASI, Psoriasis Area Severity Index. Data presented as mean ± standard deviation or number and %.

**Table 2:** Univariate logistic regression analysis.

Variable	OR [95% CI]	*p*-Value
PsA at baseline	2.83 [1.03–7.81]	**0.044**
Bio-naïve	1.85 [1.03–3.34]	**0.040**
Number of previous biological therapies	0.81 [0.64–1.02]	0.074
Anti-IL-17 as last therapy before guselkumab	0.53 [0.28–0.98]	**0.043**
Anti-TNFα as last therapy before guselkumab	0.57 [0.26–1.26]	0.164
Ustekinumab (anti-IL-12/IL-23) as last therapy before guselkumab	1.13 [0.64–2.00]	0.043
PASI score at baseline	1.01 [0.98–1.03]	0.603
Male sex	0.75 [0.44–1.29]	0.299
Age	0.99 [0.98–1.01]	0.544
Number of comorbidities	0.90 [0.74–1.10]	0.291
Age at onset	1.01 [0.99–1.02]	0.516
Duration of disease	0.99 [0.97–1.01]	0.199
Weight	0.99 [0.98–1.00]	0.304
BMI	0.97 [0.93–1.02]	0.270
Scalp involvement	1.45 [0.84–2.53]	0.185
Genital involvement	1.13 [0.62–2.06]	0.692
Nails involvement	0.77 [0.31–1.93]	0.582
Palmo-plantar involvement	0.66 [0.29–1.51]	0.324

Univariate logistic regression analysis of variables associated with super-responder status at week 20 (achievement of PASI = 0).

**Table 3:** Stepwise multivariate logistic regression model A.

Variable	OR [95% CI]	*p*-Value
PsA at baseline	2.93 [1.06–8.14]	0.039
Bio-naïve	1.90 [1.05–3.43]	0.035

Number of obs = 232; LR chi^2^ = 9.38; Prob > chi^2^ = 0.0092; Pseudo R^2^ = 0.0305.

**Table 4:** Stepwise multivariate logistic regression model B.

Variable	OR [95% CI]	*p*-Value
PsA at baseline	3.64 [1.26–10.55]	0.017
Number of previous biological therapies	0.75 [0.58–0.96]	0.023

Number of obs = 232; LR chi^2^ = 10.08; Prob > chi^2^ = 0.0065; Pseudo R^2^ = 0.0327.

**Table 5:** Stepwise multivariate logistic regression model C.

Variable	OR [95% CI]	*p*-Value
PsA at baseline	3.07 [1.09–8.65]	0.034
Anti-IL-17 as last therapy before guselkumab	0.46 [0.24–0.88]	0.019
Anti-TNFα as last therapy before guselkumab	0.42 [0.18–0.97]	0.042

Number of obs = 232; LR chi^2^ = 12.79, Prob > chi^2^ = 0.0051; Pseudo R^2^ = 0.0415.

## Data Availability

The original contributions presented in the study are included in the article; further inquiries can be directed to the corresponding author.
